# Tumour-infiltrating Langerhans cells in non-melanoma skin cancer, a clinical and immunohistochemical study

**DOI:** 10.3332/ecancer.2020.1045

**Published:** 2020-05-15

**Authors:** Alaa Maraee, Azza Gaber Antar Farag, Maram Mashhour Gadallah, Asmaa Gaber Abdou

**Affiliations:** 1 Dermatology, Andrology and STDs Departments, Faculty of Medicine, Menoufia University, Shebein Elkom, 32511, Egypt; 2 Department of Pathology, Faculty of Medicine, Menoufia University, Shebein Elkom, 32511, Egypt

**Keywords:** CD1a, non-melanoma skin cancer, Langerhans cells

## Abstract

Non-melanoma skin cancer, including basal cell carcinoma (BCC) and squamous cell carcinoma (SCC) represents 78.5% of all skin malignant tumours in Egypt. Dendritic cells can be found in almost all human tumours, they play an important role in antitumour immunity. The aim of the present study was to evaluate the percentage of Langerhans cells using CD1a in non-melanoma skin cancer, including BCC and SCC and to correlate this percentage with their clinicopathological features. The current study was performed on surgically excised specimens of 41 patients presented with non-melanoma skin cancer (26 BCC and 15 SCC) and 16 healthy volunteer control subjects. The mean and median percentage of Langerhans cells were higher in normal epidermis of control compared to malignant tumour tissue (p < 0.0001) and adjacent epidermis overlying malignant tumour tissue (p = 0.007). Langerhans cells were significantly seen in BCC cases more than SCC (p = 0.035) and they were seen in facial lesions more than those arising from other sites (p = 0.007). The reduction of Langerhans cells is a way for non-melanoma skin cancer to develop and progress. Marked reduction of Langerhans cells in SCC compared to BCC could refer to their role as a barrier against metastasis.

## Introduction

Skin cancer is the most common cancer in Europe, the United States and Australia, and basal cell carcinoma (BCC) accounts for approximately 80% of all skin cancers [1]. According to the Egyptian National Cancer Institute, 78.5% of skin cancers are non melanocytic, including BCC (54.6%) and SCC (44.9%) [2].

Dendritic cells (DC) represent a small subset of immune cells that are derived from the bone marrow and are found in nearly every tissue in the human body [3]. Langerhans cells are the dendritic cells of the epidermis that comprise 3%–5% of the total epidermal cell population [4] recognised by specific markers, such as CD207 [5] and CD1a [6]. These molecular markers also play functional roles in various aspects of Langerhans cells biology [7].

CD1a belongs to transmembrane glycoproteins (CD1) that is structurally related to the major histocompatibility complex (MHC) proteins [8]. Langerhans cells are considered to play an important role in antitumour immunity. The vaccination with dendritic cells pulsed with tumour peptides, lysates or RNA, or loaded with apoptotic and necrotic tumour cells could induce significant antitumour immunity [9].

The aim of the present study was to evaluate the percentage of Langerhans cells using CD1a in non-melanoma skin cancer, including BCC and SCC and to correlate this percentage with their clinicopathological features.

## Methods

This study was carried out on excised surgical specimens of 41 patients presented to Clinic of Dermatology and Andrology Department, Faculty of Medicine, Menoufia University with clinical features of malignant ulcer (necrotic floor, everted or rolled in edges and firm base) in the period between January 2017 and June 2019. The patients were transferred to the Surgical Department, Faculty of Medicine, Menoufia University where resection with safety margins was performed. Normal skin biopsies of normal subjects attending Plastic Surgery Department, Faculty of Medicine, Menoufia University were included as a control group. The study was approved by the ethical committee of the Faculty of Medicine, Menoufia University. Each patient was subjected to history taking, general and local examinations. Data included age, gender, size and site of lesion.

### Histopathological assessment

The specimens were sent to the Pathology Department, Faculty of Medicine, Menoufia University for routine tissue processing. Several 5 micron thick sections were cut from the prepared paraffin embedded blocks, one to be stained by hematoxylin and eosin and another sections were mounted on poly L lysine coated slides for immunohistochemistry. Hematoxylin and eosin stained slides were evaluated for diagnosis (BCC and SCC), grading of SCC (well, moderate and poor differentiation) and assessment of margins (positive and negative). Staging of SCC was performed according to American Joint Committee on Cancer 8th edition [10].

### Immunohistochemical staining

The method used for immunostaining was a streptavidin-biotin-amplified system. The antibody used was mouse monoclonal antibody, anti CD1a 7.0 ml ready to use (clone 010) (Dako, Copenhagen, Denmark). Slides were subjected to deparaffinisation and rehydration. Antigen retrieval was performed by boiling in citrate buffer saline (pH 6), followed by cooling at room temperature. Endogenous perioxidase was blocked by incubation with H_2_O_2_, 3%. The primary antibody was incubated overnight at room temperature, and then the secondary antibody (ready-to-use, Ultravision detection system anti-polyvalent HRP/DAB, Thermoscientific, Labvision, Fremont, CA, USA) was applied with DAB as a chromogenic substrate and Mayer’s hematoxylin as a counter stain. Normal skin was used as a positive control for CD1a. The replacement of the primary antibody in the staining procedure with mouse IgG1 isotype (Dako, Copenhagen, Denmark) at the same dilution of primary antibody was included as a negative control.

### Interpretation of CD1A immunostaining

Membranous immunostaining in any number of cells was required to assign CD1a positivity. The percentage of positive CD1a cells (Langerhans cells) was assessed and expressed as mean, median and range. The tumour area and adjacent normal epidermis overlying malignant tumour tissue were evaluated for the percentage of Langerhans cells. Three randomly chosen low power fields (1 LPF = 0.40 mm^2^) (Leica, Germany) were analysed for the percentage of Langerhans cell infiltrate out of total number of cells as determined by counting the haematoxylin stained cell nuclei in the tumour tissue and the tumour stroma and averaged in each case.

### Statistical analysis

Data were collected, tabulated and statistically analysed using a personal computer with the ‘Statistical Package for the Social Sciences’ (SPSS) version 22 program. Mann–Whitney test was used for evaluation of quantitative variables and Fisher’s exact and chi-square tests were used for qualitative variables. *p*  <  0.05 was considered as significant.

## Results

### The clinicopathologic features of studied cases

The current study included 41 non melanoma skin cancer composed of 26 BCC and 15 SCC, their age ranged between 16 and 91 years with a mean of 58.93 ± 15.29 and a median of 62 years. The cases were 23 males and 15 females and the facial location was the main site affected in most of cases (36, 87.8%). The size of lesion ranged between 0.3 and 15 cm with 2 cm as a median value and 3.183 ± 3.03 as a mean value. Regarding SCC, most cases were moderately differentiated (8, 53.4%) followed by poorly differentiated in five cases (33.3%) and well differentiated in two cases (13.3%). Staging of SCC showed four cases belonged to T1, five cases belonged to T2 and T3 was formed of six cases (Table 1).

### CD1a expression

**Figure 1.**
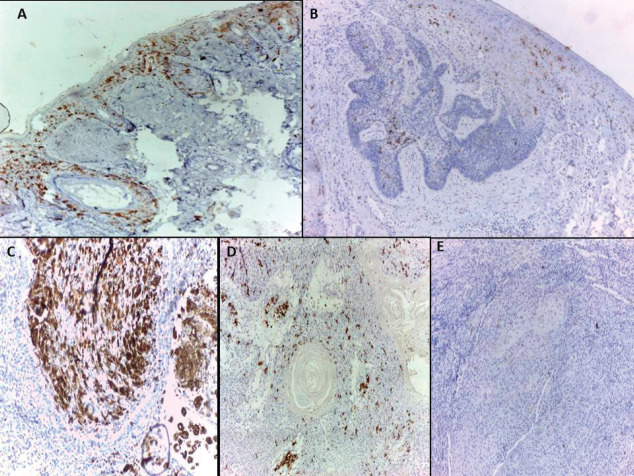
(A): Langerhans cells distributed in epidermis and hair follicles of normal skin.BCC showed few Langerhans cells (B) in one case and dense infiltrate (C) in another case. SCC showed Langerhans cells in one case (D) and their absence in another case (immunohistochemical staining ×100 for A and D, ×40 for B, ×200 for C and E).

All normal epidermis of control cases showed dendritic cells positive for CD1a (Langerhans cells) (Figure 1A) compared to its expression in peritumoural areas in 80.5% of malignant cases (Figure 1B–D) with no significant difference (*p* = 0.155). However, the mean and median percentage of Langerhans cells were higher in normal epidermis of control cases compared to malignant tumour tissue (*p* < 0.0001) and adjacent epidermis overlying malignant tumour tissue (*p* = 0.007). On the other hand, no significant differences between malignant tumour tissue and adjacent epidermis overlying malignant tumour tissue were detected as regards the percentage of Langerhans cells (Table 2).

### The relationship between presence of Langerhans cells (CD1a positive) and clinico-pathological data of malignant cases

There was a significant association between presence of Langerhans cells highlighted with CD1a and BCC compared to SCC, since more positive CD1a was seen in BCC (*p* = 0.035**)**. Furthermore, the presence of these cells was associated with the site of skin cancer, since facial location showed Langerhans cells more than other sites (extremities, trunk and others) (*p* = 0.014). Although the association was not significant, large tumour size was associated with absence of Langerhans cells compared to small sized tumours and most cases with free surgical margins (69.7%) showed Langerhans cells (Table 3). On the other hand, the percentage of Langerhans cells did not show any statistical association with other studied features (data not shown).

## Discussion

DCs can be found in almost all human tumours, and their ability to take up antigen and initiate an aggressive immune response makes them attractive targets for cancer immunotherapies. Moreover, while the immune system has the innate ability to recognise and attack cancer cells, tumours often evade detection by downregulating antigen presentation and impairing DC function [11]. The effective restoration of DC activity may, therefore, prove critical in successful tumour detection and the generation of a potent antitumour response.

The present study used CD1a to highlight Langerhans cells (LCs) because several studies demonstrated its specificity to distinguish LCs from other DC subsets similar to Langerin (CD207) [12, 13], especially in the epidermis, however, Langerin [14] as well as CD1a [15] were reported to be expressed on a population of dermal/ connective tissue DCs, which were unrelated to LCs. Our study demonstrated lower percentage of LCs in malignant skin cases compared to normal epidermis of control agreeing with Shevchuk *et al* [7]. The regressive neoplasm of the skin had the greatest dendritic cell infiltration compared to progressive neoplasm [16]. Furthermore, a decline in LCs in the epidermis above primary melanoma has been reported [17] together with a significant decline in the numbers of LCs in deeply invasive human melanomas [18] suggesting that a decline in LC numbers favours persistence of the melanoma. It was also observed that such decrease in dendritic cell number could be a bad prognostic factor for other solid tumours as well [7].

Tumours are thought to impair antigen presentation and the establishment of a tumour-specific immune response through a variety of mechanisms. For instance, tumour cells often secrete IL-6 and macrophage colony-stimulating factor, which may shift the differentiation of monocytes towards macrophages rather than DCs. This effectively inhibits the priming of tumour-specific T cells [19]. Furthermore, tumour cells may interfere with DC maturation through the secretion of IL-10, which results in the induction of antigen-specific energy [20].

Langerhans cells density was proposed as a prognostic marker for laryngeal squamous cell carcinomas [21] and breast cancer [22]. Moreover, the lack of CD1a expression in the dendritic cells of Barrett’s mataplasia may predict its evolution toward esophageal adenocarcinoma [23].

The present study demonstrated and confirmed the presence of LCs in normal epidermis where they are generally found in the basal and supra-basal layers forming a dense network of cells together with follicular and interfollicular regions [24, 25]. The higher percentage of LCs in normal epidermis encountered in the present study compared to other studies [4, 24, 25] could be due to occasional absence of hematoxylin counterstained epidermal keratinocytes nuclei within the given section plane, thereby making LC percentages apparently higher. LCs are often thought to be the first immune cells to encounter tumour antigens from cutaneous cancers. Initiating tumour immunity may, therefore, be critically dependent on the proper functioning of DCs as antigen presenters, with the ability to stimulate T cell proliferation and polarisation. The present study demonstrated less number of SCC cases that showed tumour associated LCs compared to BCC. Previous reports have shown reduced quantities of both LCs and CD11c+ dermal DCs in SCC lesions indicating a disruption in DC generated immunity [26, 27].

Furthermore, tumour-associated mDCs were poor stimulators of T cell proliferation when compared to their peritumoural or healthy skin counterparts. Tumour-associated mDCs extracted from BCC lesions have also been shown to be deficient activators of the T cell response when compared to normal cutaneous mDCs [28]. Some studies demonstrated no significant differences in the number of LCs in human BCC skin lesions relative to normal skin; however, an increased number of LCs has been found in the epidermis adjacent to the tumour [29, 30]. The higher numbers of adjacent LCs were associated with a lower potential for aggressiveness of the tumour, suggesting that LCs might play a role in limiting tumour growth [31].

Experimental depletion of LCs in SCC induced in a mouse model results in acceleration of tumour growth during the 12 weeks of the study suggesting that LCs have a tumour suppressive effect during the initiation phase of cancer development [32]. On the other hand, presence of CD1a expressing cells in tumours may influence metastasis [33].

Although not reaching statistical significance, the current study demonstrated that Langerhans cells were associated with small sized tumours and free surgical margins. This emphasised on the importance of Langerhans cells as a weapon against skin cancer progression and development.

## Conclusions

The reduction of Langerhans cells is a way for non-melanoma skin cancer to develop and progress. Marked reduction of Langerhans cells in SCC compared to BCC could refer to their role as a barrier against metastasis.

## Conflicts of interest

The authors declared no conflict of interests.

## Funding

This study was not funded by any resources.

## Figures and Tables

**Table 1.** The clinico-pathological data of studied cases (*n* = 41).

Clinico-pathological data	Distribution	
	No.	%
Age (years) : Mean ± SD Median Range (Min. – Max.)	58.93 ± 15.291 62 16–91	
Gender: Male Female	23 18	56.1 43.9
Male:female ratio	1.28:1	
Type of tumour: BCC SCC	26 15	63.4 36.6
Site of tumour: Facial Trunk (vulva) Extremities (sole) Extremities (Leg)	36 3 1 1	87.8 7.4 2.4 2.4
Size of tumour (cm): Mean ± SD Median Range (Min. – Max.)	3.183 ± 3.03 2 0.3–15	
Margin: Free Involved	29 12	70.8 29.2
Grade of SCC (*n* = 15): Well differentiated Moderate differentiated Poorly differentiated	2 8 5	13.3 53.4 33.3
Stage of SCC (*n* = 15): T1 T2 T3	4 5 6	26.7 33.3 40

**Table 2.** Comparison between Malignant, adjacent epidermis overlying tumour and epidermis of control groups regarding CD1a data.

CD1a data	Malignant No. (%)	Adjacent epidermis overlying tumour N (%)	Control No. (%)	Test of significance
CD1a status Positive Negative	33 (80.5) 8 (19.5)	33 (80.5) 8 (19.5)	16 (100) 0 (0)	P1=0.09# **P2--------** P3=0.09#
CD1a percentage Mean ± SD Median Range	11.9±13.59 10 0-50	14.63±11.09 15 0-30	24.38±7.93 20 15-40	P1 < 0.001** @ P2= 0.217@ P3=0.007*@

#: Fischer’s exact test, @: Mann Whitney test **: highly significant, *: significant.

P1; malignant versus control, P2 ;malignant versus adjacent, P3 ;adjacent versus control.

**Table 3.** The relationship between CD1a and clinico-pathological data of malignant cases.

clinico-pathological data	CD1a		Test of significance	*p* value
	Positive (*n* = 33) No (%)	Negative (*n* = 8) No (%)		
Age: Mean ± SD Median Range	59.27 ± 14.25 62 16–80	57.5 ± 20.11 60 28–91	*U*** =** 0.695	0.487
Gender: Male Female	19 (57.6) 14 (42.4)	4 (50) 4 (50)	FE = 0.15	0.713
Type of tumour: BCC SCC	24 (72.7) 9 (27.3)	2 (25) 6 (75)	FE = 6.332	0.035*
Site of tumour: Facial Others	31 (93.9) 2 (6.1)	5 (62.5) 3 (37.5)	*X***^2^=** 5.94	0.014*
Size of tumour (cm): Mean ± SD Median Range	2.703 ± 2.24 2 0.3–10	5.163 ± 4.89 3.25 0.8–15	*U*** =** 1.47	0.142
Margin: Free Involved	23 (69.7) 10 (30.3)	6 (75) 2 (25)	*X*^2^ = 0.09	0.77
Grade of SCC: Well differentiated Moderate differentiated Poorly differentiated	**(*n* = 9)** 1 (11.1) 5 (55.6) 3 (33.3)	**(*n* = 6)** 1 (16.7) 3 (50) 2 (33.3)	*X*^2^ = 0.1	0.95
Stage of SCC: T1 T2 T3	(*n* = 9) 3 (33.3) 3 (33.3) 3 (33.3)	(*n* = 6) 1 (16.7) 2 (33.3) 3 (50)	*X*^2^ = 0.63	0.73

FE: Fischer exact test, *X***^2^**: chi square, U: Mann–Whitney, *: significant.
